# Frozen Vegetable Processing Plants Can Harbour Diverse *Listeria monocytogenes* Populations: Identification of Critical Operations by WGS

**DOI:** 10.3390/foods11111546

**Published:** 2022-05-25

**Authors:** Pilar Truchado, María I. Gil, Ania Pino Querido-Ferreira, Cecilia López Capón, Avelino Álvarez-Ordoñez, Ana Allende

**Affiliations:** 1 Research Group on Microbiology and Quality of Fruit and Vegetables, CEBAS-CSIC, 30100 Murcia, Spain; migil@cebas.csic.es (M.I.G.); aallende@cebas.csic.es (A.A.); 2 CETAL, Centro Tecnológico Agroalimentario de Lugo, 27002 Lugo, Spain; ania@allgenetics.eu (A.P.Q.-F.); celop4@yahoo.es (C.L.C.); 3 Department of Food Hygiene and Technology, Universidad de León, 24007 León, Spain; aalvo@unileon.es; 4 Institute of Food Science and Technology, Universidad de León, 24071 León, Spain

**Keywords:** environmental monitoring, whole genomic sequencing, freezing plants, food safety, microbial risk

## Abstract

Frozen vegetables have emerged as a concern due to their association with foodborne outbreaks such as the multi-country outbreak of *Listeria monocytogenes* serogroup IVb linked to frozen corn. The capacity of *L. monocytogenes* to colonize food-processing environments is well-known, making the bacteria a real problem for consumers. However, the significance of the processing environment in the contamination of frozen foods is not well established. This study aimed to identify potential contamination niches of *L. monocytogenes* in a frozen processing plant and characterize the recovered isolates. A frozen vegetable processing plant was monitored before cleaning activities. A total of 78 points were sampled, including frozen vegetables. Environmental samples belonged to food-contact surfaces (FCS); and non-food-contact surfaces (n-FCS). Positive *L. monocytogenes* samples were found in FCS (*n* = 4), n-FCS (*n* = 9), and the final product (*n* = 1). A whole-genome sequencing (WGS) analysis revealed two clusters belonging to serotypes 1/2a-3a and 1/2b-3b). The genetic characterization revealed the presence of four different sequence types previously detected in the food industry. The isolate obtained from the final product was the same as one isolate found in n-FCS. A multi-virulence-locus sequence typing (MVLST) analysis showed four different virulence types (VT). The results obtained highlight the relevant role that n-FCS such as floors and drains can play in spreading *L. monocytogenes* contamination to the final product.

## 1. Introduction

*Listeria monocytogenes* is a human pathogen that is widely present in the environment (soil, water, and organic material) and, unlike most bacteria, can grow and multiply at low temperatures [1,2]. *L. monocytogenes* could represent a serious public health risk. The relevance of this foodborne pathogen in frozen vegetables in Europe (EU/EEA) is evidenced by the number of foodborne outbreaks and notifications to the Rapid Alert System for Food and Feed (RASFF) linked to *L. monocytogenes*, where frozen vegetables were implicated [3]. One of the most relevant foodborne outbreaks was a multi-country outbreak of *L. monocytogenes* ST6 linked to frozen corn that caused 53 cases and 10 deaths over the period 2015–2018 [4]. The detection of this outbreak was possible thanks to the use of whole-genome sequencing (WGS), which facilitated the linkage between human and food isolates. The outbreak investigation concluded that the environmental contamination of a frozen vegetable plant in Hungary was the source of the strain causing the outbreak [5]. This finding suggested that the strain had persisted in the processing plant despite the cleaning and disinfection procedures that were carried out. It was also highlighted that some frozen fruits and vegetables can be added uncooked to ready-to-eat salads or used in smoothies or other products without being subjected to any process to eliminate or reduce the level of pathogens, increasing the risk of listeriosis [3]. 

Several studies have demonstrated the capacity of *L. monocytogenes* to colonize food processing environments, making this hazard a serious problem for food business operators and consumers due to the risk of contamination of the final product [6,7]. *Listeria monocytogenes* can to adhere to biofilms on industrial surfaces, especially where water and organic residues accumulate, being more difficult to eradicate and therefore to control [8]. Many produce outbreaks and recalls due to *L. monocytogenes* are traced back to the processing environment [9]. The ability of *L. monocytogenes* to grow and spread at low temperature conditions and its tolerance to stress conditions such as pH and salinity not only contributes to its ubiquitous nature but also to its persistence in food processing environments and high risk of cross-contamination [10,11]. The capability of *L. monocytogenes* to persist in inaccessible surfaces that are difficult to clean favours the exposure of the bacteria to dilute subinhibitory concentrations of sanitizers [12,13]. In addition, temporal breakdowns in hygiene barrier efficiency, poor hygiene practices and unhygienic design of equipment may trigger *L. monocytogenes* food plant contamination.

*L. monocytogenes* strains have been classified into four different lineages. The most widely spread ones are Lineage I, including serotypes 1/2b and 4b, which are frequently associated with human listeriosis cases, and Lineage II, including serotypes 1/2a and 1/2c, which are more commonly found in food and food processing environments [11]. *L. monocytogenes* can also be classified into 13 different serotypes based on somatic and flagellar antigens, being the above-mentioned serotypes (1/2b, 4b, 1/2a and 1/2c) responsible for 90% of worldwide listeriosis outbreaks [14,15].

Pulsed field gel electrophoresis (PFGE) is one of the most commonly used subtyping methods to classify *L. monocytogenes* strains, particularly to determine persistent strains in food processing facilities, sources of contamination, and transmission routes [16,17]. More advanced molecular-based techniques, such as WGS, allow for the discrimination of *L. monocytogenes* strains down to single nucleotide differences, providing an accurate characterization of strains and tracking the causes of outbreaks. During the last few years, the use of WGS in combination with bioinformatics tools in outbreak investigations has helped find the attribution source of infections, evidencing their power as typing tools in epidemiological surveillance [18,19,20,21,22]. However, until now, only a few studies have used WGS for the assessment of *L. monocytogenes* population diversity in food processing plants. The main drawbacks of WGS are the high costs and the complexity of analysis, which make it necessary to rely on highly qualified staff for the interpretation of the data. However, the use of WGS in the food processing environment represents a powerful tool to enhance the understanding of the origin, cross-contamination, reservoir, and possible persistence of specific subpopulations along the food chain [11,23].

Knowing the capacity of *L. monocytogenes* to persist in the environment and taking into account current consumer practices that consume frozen fruit and vegetables as ready to eat (RTE) products, it is important to identify potential contamination sources of the pathogen in the environment of frozen processing plants. This study includes an overview of the most critical steps that could be considered when establishing a routine monitoring program for *L. monocytogenes* in frozen vegetables and their food processing environment. Therefore, the main objective was to monitor the colonization by *L. monocytogenes* of a frozen vegetable processing plant and characterize by WGS all the isolates recovered to obtain insights into the prevalence of this important foodborne pathogen in industrial settings.

## 2. Materials and Methods

### 2.1. Sampling

Systematic environmental sampling was performed in a frozen vegetable plant located in Murcia (Spain). Sampling was carried out after the production of frozen cut peppers, just before sanitation. In the environmental monitoring (EM), a total of 78 points were sampled including food contact (FCS) and non-food contact surfaces (n-FCS). Additionally, samples from raw materials, the final product and process water were also collected. Sterile cotton swabs (Deltalab, Barcelona, Spain), pre-moistened with sterile water, were used for sampling surfaces and areas that were difficult to reach. At each sampling point, swabs were dragged back and forth to cover the whole surface. Swabs were added to sterile tubes with 10 mL of half Fraser broth (Scharlab; Barcelona, Spain). Three samples of raw material (100 g each), the final product (100 g each) and process water samples (2 L each) collected in sterile conditions were also taken. All samples were kept at approx. 4 °C in a cooling box containing ice packs and transported to the CEBAS-CSIC laboratory within one-hour samples were processed.

### 2.2. Detection and Isolation of Listeria monocytogenes Strains

The analysis of samples was performed following the specific ISO standard method (EN ISO 11290-1) with some modifications. The presence/absence of *L. monocytogenes* was assessed after two enrichment steps in Half Fraser Broth and Fraser Broth (Scharlab), respectively. Samples of raw material and final produce (25 g) were homogenized with 225 mL of half-Fraser broth (Scharlab). Water samples were filtered through 0.45 µm membrane filters (Sartorius, Madrid, Spain) using a filter holder manifold (Millipore, Madrid, Spain), and the filters were incubated with 100 mL of Half Fraser Broth [3,4]. In the case of swabs, they were immersed in 10 mL of half Fraser broth (Scharlab). All samples were incubated at 30 °C for 24 h. Then, 0.1 mL of each pre-enrichment was added to 10 mL of Fraser Broth and the secondary enrichment tubes were incubated at 37 °C for 48 h. 

The secondary enrichment samples were streaked onto ALOA/OCLA Listeria selective agar (Scharlab) plates which were incubated for 24 h at 37 °C before the interpretation of the results. The presumptive *L. monocytogenes* strains, forming blue colonies with an opaque halo, were analysed using a *L. monocytogenes* GeneDisc Pack in a Genedisc Cycler multiplex PCR (Pall^®^ Corporation, Washington, WA, USA) following the manufacturer’s instructions after the extraction of DNA. Positive *L. monocytogenes* isolates after the Gene Disc Pack were submitted to identification by MALDI-TOF mass spectrometry, using the Biotyper platform (Bruker Daltonics, Bremen, Germany) as previously described in Truchado et al. [24]. *L. monocytogenes* isolates confirmed by the MALDI-TOF mass spectrometry were frozen in brain-heart infusion broth (BHI, Scharlab), with 30% glycerol, at −80 °C.

### 2.3. Whole Genome Sequencing

#### 2.3.1. Library Preparation

Genomic DNA from confirmed *L. monocytogenes* isolates was purified using the Gentra Puregene Yeast/Bacterial Kit (Qiagen, Hilden, Germany). DNA was quantified by Qubit 3.0 (Life Technologies, Carlsbad, CA, USA) and stored at −20 °C until library preparation. DNA libraries were prepared using the Ion Xpress Plus Fragment Library kit using the enzymatic fragmentation procedure following the manufacturer’s instructions (Life Technologies, ThermoFisher Scientific, Carlsbad, CA, USA). Each library was indexed using Ion Xpress™ Barcode Adapters (Life Technologies, ThermoFisher Scientific). Ion Library TaqMan Quantitation Assay for qPCR (7500 Fast, Life Technologies, ThermoFisher Scientific) was used for library quantification. Template preparation was performed using the Ion OneTouch™ 2 System and the Ion PGM™ Hi-Q™ View OT2 400 Kit (Life Technologies, ThermoFisher Scientific). The libraries were sequenced with the Ion Torrent PGM sequencer (Life Technologies, ThermoFisher Scientific) using the Ion PGM™ Hi-Q™ View Sequencing Kit (Life Technologies, ThermoFisher Scientific), following the manufacturer’s protocols. To obtain deep sequencing results for each sample, seven barcoded samples were sequenced in each Ion chip 318 (Life Technologies, ThermoFisher Scientific) with more than 40× coverage per sample.

#### 2.3.2. Quality Assurance and Genome Assembly

The quality of the reads was checked with FastQC v.0.11.2 [25] on raw sequencing data. Low-quality sequences were trimmed using Trimmomatic v. 0.32 [26]. The assembly of trimmed reads was performed using the Pilon Galaxy version in October 2019 [27], and the strain *L. monocytogenes* NC_003210.1 as the reference genome. The evaluation of genome assemblies was performed by QUAST (Galaxy version 5.0.2 + Galaxy1) [28]. Samples quality control (QC) statistics were analysed with Ridom SeqSphere+ version 6.0.2 [29].

#### 2.3.3. Serotyping, MLSTs, Core Genome MLST, Phylogenomic Analysis and MVLST

For each isolate, the *L. monocytogenes* 5-plex PCR Serogroup task templates of the Ridom SeqSphere version 6.0.2 software [26] with fragments from five DNA regions (lmo118, lmo0737, ORF2110, ORF2829, and prs as an internal amplification control) were developed for molecular separation of the four major serotypes (1/2a, 1/2b, 1/2c, and 4b) [30]. Additionally, sequencing reads were mapped against a Multi Locus Sequence Typing (MLST) scheme based on seven housekeeping and virulence genes developed by the Institute Pasteur [23] to identify Sequence Types (STs) and Clonal Complexes (CCs). The Galaxy Aries platform was used for the verification of serotypes (LisSero v1.0) and MLST types (MLST List v 2.16.1 and MentaLiST MLST Analysis 0.2.3). The core genome MLST (cgMLST) analysis was performed by using MLST+ Target Definer (version 1.1.), composed of 1704 targets [31] to identify the complex types (CTs). Calculation of allelic distances and minimal spanning trees was performed using the in-built distance matrix and minimum spanning tree functions [32]. The Multi Virulence Locus Sequence Types (MVLSTs) were determined in silico by using the scheme described by Zhang et al. [33].

## 3. Results

### 3.1. Identification of Listeria monocytogenes Strains

A total of 78 samples were collected from the commercial frozen vegetable processing plant. Sampling points were divided into zones based on the FDA draft guidance on *L. monocytogenes* control in ready-to-eat foods [34]. A total of 32 samples were taken from FCS, while 37 samples were obtained from n-FCS close to the processing area and directly adjacent to FCS (e.g., exterior of equipment, framework, food carts, equipment housing, gears, ventilation/air handling equipment, and floors). Table 1 summarizes the type and number of samples collected in each zone.

**Figure 1:**
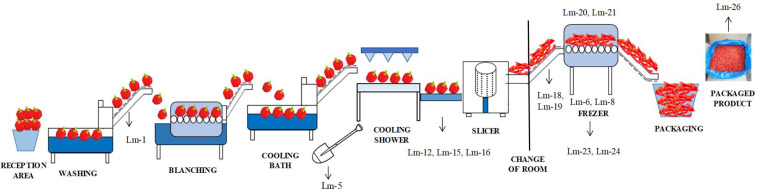
Scheme of sampling positive points for *L. monocytogenes*.

Among the 78 samples taken from the environment and frozen product, the presence of *L. monocytogenes* was initially confirmed in 34 samples after the enrichment and culture growth in selective ALOA/OCLA media. A multiplex- PCR Gene disc identified 27 out of the 34 isolates as *L. monocytogenes* (Table 2). Further confirmation by matrix-assisted laser desorption ionization time-of-flight (MALDI-TOF) mass spectrometry (MS) confirmed 14 isolates as *L. monocytogenes* (Table 2). Expectedly, the prevalence of *L. monocytogenes* was higher in n-FCS (24.3%) than in FCS (12.5%). Figure 1 shows an overall view of the sampling points where *L. monocytogenes* were detected within the processing line and the isolate enumerated. Among the FCS, *L. monocytogenes* were found in conveyor belts (Lm-1), the shovel used to move the product from the cooling bath to the conveyor belt (Lm-2) and the freezing tunnel (Lm-3 and Lm-4). Among the n-FCS sampling points, *L. monocytogenes* was found mostly in the floors and drains, particularly in the drains placed around the conveyor belt for manual inspection, as well as in the freezing tunnel and the surrounded area (Lm8 to Lm13) (Table 1 and Figure 1).

### 3.2. Identification of Serotypes, Multi Locus Sequence Typing and Multi-Virulence-Locus Sequence Typing Analyses by Whole Genome Sequences

The *L. monocytogenes* isolates (14) found in FCS and n-FCS, as well as in the final product, were subjected to molecular serotyping by WGS using the function Serotyping 5-PCR Plex and QC included in the Ridom SeqSphere software version 6.0.2 [29]. The results showed that the isolates belonged to the phylogenetic lineages I and II (Table 3). From the isolates, 64.3% (9/14) belonged to serotype 1/2a-3a and 35.7% (5/14) to serotype 1/2b-3b-7. Other relevant serotypes from linages I and II, such as 1/2c-3c, 4b-4d-4e or 4a-4c, were not detected. These results were confirmed using the LisSero tool [30] implemented in the Galaxy Aries platform.

Based on the sequences obtained, four sequence types (STs) and clonal complexes (CCs) were found among the tested strains (Table 3). The most abundant ST/CC were ST7/CC7 (57%), followed by ST87/CC87 and ST5/CC5 (14%), and ST8/CC8 (7%). Only one strain isolated from the floor located below the conveyor belt used for manual inspection (Lm 12) could not be assigned to a known ST/CC by the MLST analysis. When the Ridom SeqSphere+ software was used for assembly and analysis of the cgMLST data using the SeqSphere+ Pipeline Mode, three different CTs were detected in 5/14 samples including 3714 (Lm-12), 7746 (Lm-6 and Lm-8) and 6480 (Lm-18 and Lm-20). The isolates corresponding to CT 7746 were isolated from FCS sample points obtained from the freezing tunnel, while the isolates corresponding to CT 6480 were obtained from samples belonging to n-FCS from the floor located close to the conveyor belt before the freezing tunnel. On the other hand, the isolate corresponding to CT 3714 was obtained from the floor placed near the conveyor belt used for manual inspection.

**Figure 2:**
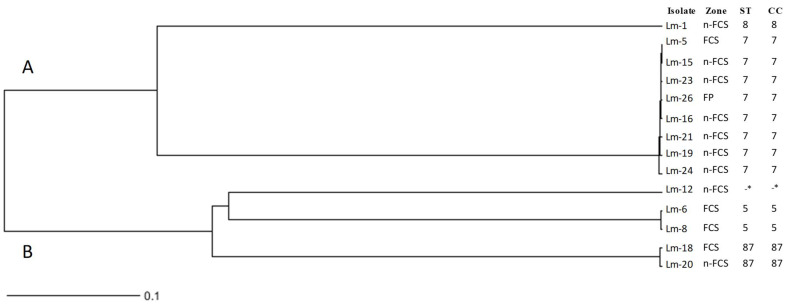
Dendrogram showing the relationships among the 14 *L. monocytogenes* isolates obtained from the final product (FP), FCS and n-FCS of the frozen vegetable plant. The dendrogram was generated using the unweighted pair group method with averages (UPGMA). Information on the isolate identification, sampling zone, sequence type (ST) and the unique clonal complex (CC) of each *L. monocytogenes* strain is shown. -* Unknown.

Cluster and Minimum Spanning Tree (MST) analyses were elaborated to show the relatedness of the 14 tested strains obtained from the different zones of the frozen vegetable processing environment. The cluster analysis revealed that strains were distributed in two clusters in terms of serotypes (A and B) (Figure 2). Cluster A included two sub-clusters, one composed of all isolates identified as ST7/CC7 (*n* = 8), and a second cluster formed by the isolate identified as ST8/CC8 (Lm-1). Cluster B is also formed by two sub-clusters, one including the *L. monocytogenes* isolate identified as ST87/CC87 (CT6480), and one, which is also divided into two more groups, including the isolate from an unknown ST (CT3714) and the strains identified as ST5/CC5 (CT7746).

**Figure 3:**
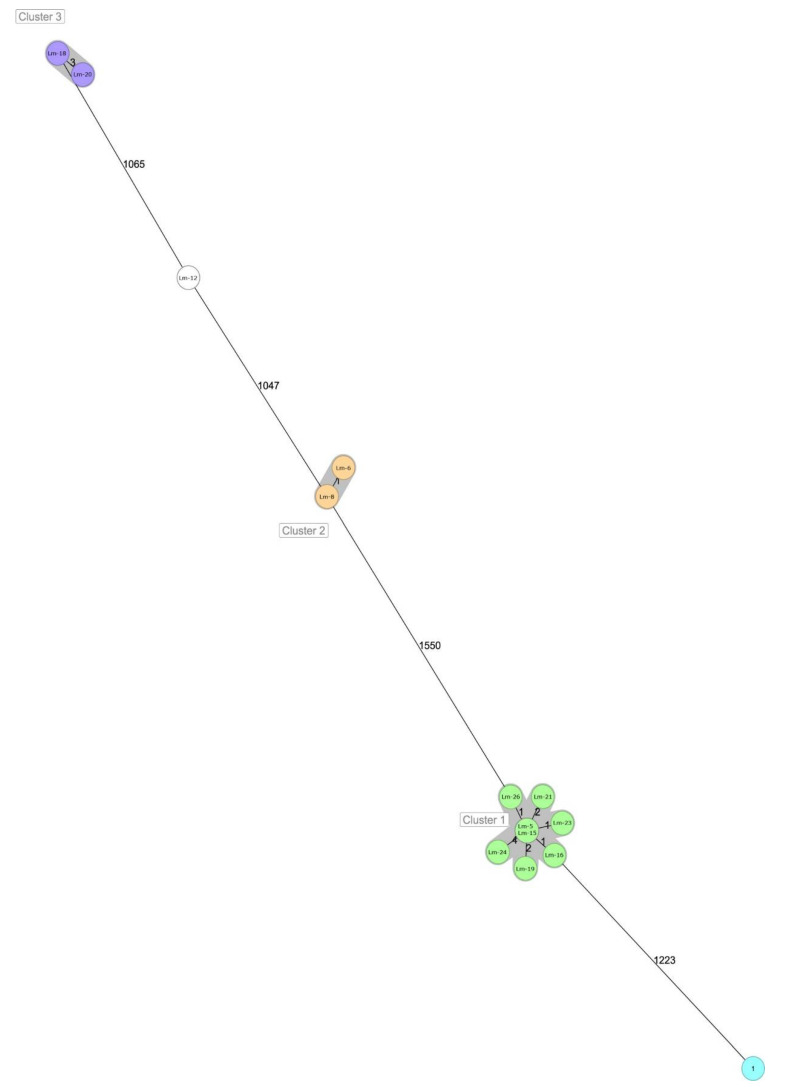
Minimum spanning tree (MST) showing the relatedness of the 14 isolates of *L. monocytogenes* strains isolated from the final product (FP),FCS and n-FCS of the frozen vegetable plant. MLST profiles are represented by circles and the colours correspond to different clusters. The length of the lines connecting MLST profiles is proportional to the number of allelic differences between circles. A grey zone surrounds the group of circles that share the same cgMLST type (STs).

On the other hand, the Minimum Spanning Tree based on the cgMLST grouped eight isolates within cluster 1. Cluster 2 contained two isolates and cluster 3 also contained two isolates (Figure 3). Table 4 shows the distance matrix constructed with differences in allelic distance between 14 strains used to build the cgMSLST.

**Figure 4:**
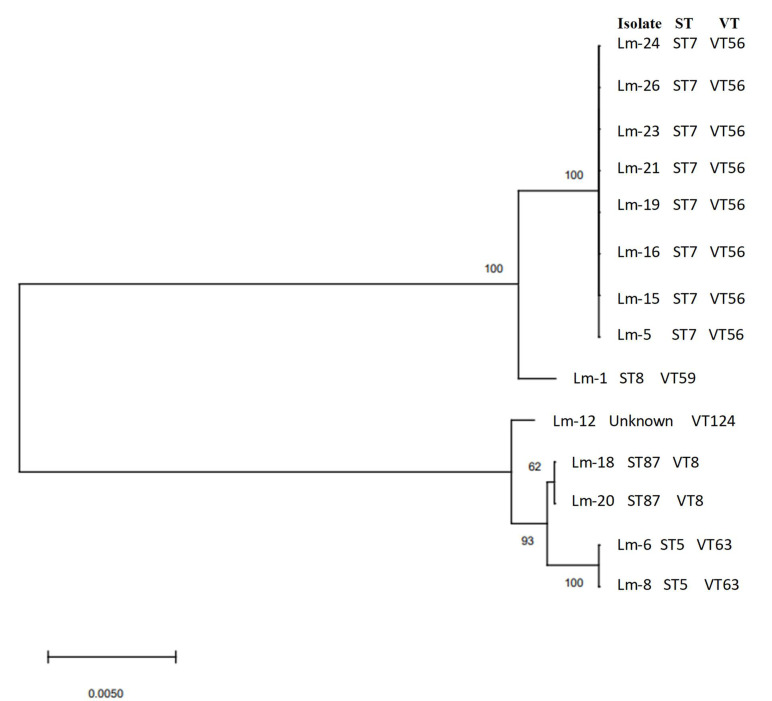
Unrooted neighbour-joining tree of the fourteen *L. monocytogenes* isolates obtained from the final product (FP), FCS and n-FCS of the frozen vegetable plant based on a multi-virulence-locus sequence typing (MVLST) analysis. Information on the sequence type (ST) and virulence type (VT) of each *L. monocytogenes* isolate is shown.

In a multi-virulence-locus sequence typing (MVLST) analysis, four virulence types (VTs) were identified (VT8, VT56, VT59, and VT63). All sequenced ST7 isolates belonged to virulence type VT56, while sequenced ST5 isolates belonged to virulence type VT63. The isolate identified as ST8 was linked to VT59 and the two isolates identified as ST87 to VT8 (Figure 4).

## 4. Discussion

The presence of *L. monocytogenes* in the environment of any food processing plant represents a serious threat to the consumers, who might be exposed through contaminated food products to the hazardous bacterium. Frozen vegetables are traditionally consumed cooked. However, consumer trends are moving towards healthier recipes, which have increased the proportion of frozen vegetables consumed raw without a cooking kill step to ensure microbiological safety [3]. This fact makes the contamination of the processing environment with this pathogen a very important concern. This was exactly the case of the multi-country outbreak of *L. monocytogenes* in frozen vegetables, which was linked to the freezing tunnel of a frozen vegetable processing plant [3,5]. The colonization by *L. monocytogenes* of a frozen vegetable processing plant was monitored and the *L. monocytogenes* isolates were characterized by WGS. As found in previous studies performed in frozen processing plants [35], positive samples for *L. monocytogenes* were more often found in n-FCS sampling points than in FCS. The contamination points where *L. monocytogenes* were detected in this study were in agreement with critical points previously identified in the frozen vegetable processing environment [3]. Places with high humidity and low temperatures such as the cooling bath and freezing tunnel areas, including the floors and drains, have been identified as ideal harbourage sites for *L. monocytogenes* [3,12,36,37], highlighting the remarkably high number of *L. monocytogenes* isolates detected from environmental sampling points associated with the freezing tunnel (36.4% (8/22)). The prevalence was much higher than that observed for any of the processing areas of the freezing plant. Resources and efforts should be focused on eliminating the contamination from this hot spot.

In the current study, *L. monocytogenes* isolates obtained from the 78 different sampling points mostly belonged to serotype 1/2a-3a, followed by 1/2b-3b-7. Serotype 1/2a-3a was often present in the floors, drains and other non-food contact samples, while 1/2b-3b-7 isolates were most frequently detected in food contact samples (conveyor belt and surfaces of the freezing tunnel). This is in agreement with previous results found in a frozen vegetable processing plant [38]. The serotypes found in the processing environment of this frozen vegetable processing plant pose a public health concern, as they have been associated with both foodborne outbreaks, as well as sporadic cases of human listeriosis [39,40]. The serotypes 1/2a, 1/2b and 4b are responsible for over 95% of the cases of listeriosis in humans, of which 1/2a and 1/2b have been mostly isolated from food and 4b from clinical cases [39,40]. In agreement with our results, the serotypes 1/2a, followed by 1/2b and 4b, have been isolated from frozen vegetables in different countries such as Spain, Switzerland and Chile [41,42,43]. In contrast, Braga et al. [14] published that serotypes 1/2b and 4b were the most frequently detected in frozen foods, though frozen vegetables were not included in that study. In general, surveillance studies in numerous geographical locations, covering several types of food (including vegetables) and environmental samples, have consistently found that 1/2a is the most common serotype [44].

The diversity of *L. monocytogenes* strains isolated from food production environments such as the meat, fish, dairy and vegetable sectors has been explored in recent years [21,45]. However, few studies have evaluated the prevalence and diversity of *L. monocytogenes* in the environment of frozen vegetable processing plants, and none on processing plants before cleaning and disinfection. In the current study, WGS-based MLST analysis showed that the most frequent STs among the isolates collected were ST7/CC7 (8/14; 57%), ST5/CC5 (2/14; 14%), ST87/CC87 (2/14; 14%) and ST8/CC8 (1/14; 7%). These *L. monocytogenes* clones have been previously identified in the environments of food processing plants. Recently, ST5 has been isolated from RTE meat processing plants in China and Poland [46,47]. Manso et al. [48] isolated ST5, ST7 and ST87 from the food processing environments of a dairy plant in samples such as floors, drains, boxes and food products. Knudsen et al. [45] identified ST7 and ST8 as persistent strains in the environment of a smoked fish processing plant. ST8 has been also identified as a persistent strain in salmon and poultry processing facilities in Europe [49,50]. Chau et al. [51] also detected the occurrence of ST87 strain in packed salmon products over a 4-year sampling period, suggesting a potential persistent contamination issue at the level of production. Moreover, strains of ST8 and ST87 have been identified as persistent contaminants in a mushroom production facility [52]. Some of the strains isolated in this study have been previously associated with disinfectant tolerance to the main sanitizers used in the food industry. Muhterem-Uyar et al., [8] identified *Listeria monocytogenes* ST5 isolated from a cheese processing environment as harbouring pLM80 plasmid with an efflux pump system (*bcrABC* cassette) and heavy metal resistance genes, which possibly have a higher tolerance to disinfectants. Knudsen et al. [45] described quaternary ammonium compound resistance-related genes (*lde* and *mdrL*) present in ST88 and ST7 strains isolated from two smoked fish processing plants, as shown by their genome wide analyses. Recently, Sun et al., [52] observed that ST8 and ST87 persistent strains isolated from a mushroom production facility were associated with biocide tolerance. However, the identification of disinfectant tolerance genes and disinfectant susceptibility was not performed in strains isolated in this study.

In the current study, one sample corresponding to unpacked frozen cut peppers was positive for *L. monocytogenes*. The isolate (Lm-26) was classified as a ST7/CC7 strain. The same serotype and complex clone were consistently found in positive samples obtained from the floors and drains (Lm-15, Lm16, Lm 23 and Lm 24). It is important to highlight that MST showed that the difference among the isolates varies between 1 and 4 alleles, which suggests that all the isolates come from the same strain (Figure 3).

Although all the samples taken from the raw material were negative for *L. monocytogenes*, it is well-known that this bacterium is frequently found in fruit and vegetables. Therefore, there is a high probability that *L. monocytogenes* contamination would have been introduced in the processing plant by the raw material. However, once in the production environment, *L. monocytogenes* might persist and cross-contaminate different product batches. The results obtained from the drainage placed close to the final product sample, where the same *L. monocytogenes* serotype was detected, indicate that the most probable origin of the contamination of the final product is from an n-FCS. As previously stated by different authors, *L. monocytogenes* present in floors and drains could potentially be moved to FCS, thereby contaminating the final products [36,37]. This finding confirmed that cross-contamination from harbourage sites to frozen vegetables was possible [10,36]. Previous studies have shown that floors, drains and other n-FCS are typical harbourage sites of *L. monocytogenes* in food production facilities and can be the origin of the spread of the pathogen, being identified as areas of high concern in food processing environments [11,12,38]. Therefore, these mentioned sites represent niches of *L. monocytogenes* in this vegetable freezing plant. However, one of the limitations of this study is that the processing plant was not monitored after cleaning and disinfection activities, preventing us from evaluating the transient or persistent nature of the isolates detected.

Based on the results obtained, further efforts should be made to intensify hygiene measures in this vegetable freezing plant. In addition, a well-designed extensive sampling programme combined with the detailed genetic characterization of isolates would help to take corrective actions to prevent the transfer of this pathogen from the environment to frozen vegetables. Regarding MVLST results, all sequenced ST7 isolates belonged to virulence type VT56, whereas all ST5 belonged to VT 63, ST8 to VT59 and ST87 to VT8. These associations ST-VT were previously described by Zhang et al. [53]. However, additional investigations should be performed to confirm whether these ST-VT associations can be considered universal.

In the sampling of frozen vegetables and processing environments of the freezing plant included in this study, *L. monocytogenes* multi-locus ST6 was not found. This ST6 was associated with listeriosis cases caused by multiple frozen foods and was also identified from surface swabs from a single plant in Hungary where vegetables were processed [5]. Nevertheless, it should be emphasized that some of the strains isolated from the frozen vegetable processing plant in the present study have been previously associated with listeriosis cases. For example, *L. monocytogenes* belonging to the serotypes ST7, ST8 and ST87 have been recently associated with listeriosis cases in China [54]. *Listeria monocytogenes* ST7 strain was responsible for an Italian outbreak associated with the pork production chain from May 2015 to March 2016, and a listeriosis outbreak also in May 2018 [55]. The serotype ST87 has been considered a potential health risk to consumers, mostly because it has been detected in clinical cases associated with the consumption of RTE foods in China [56]. ST/CC87 has been previously associated with an outbreak involving 27 human listeriosis cases in Spain in 2013–2014 [57].

## 5. Conclusions

The current study demonstrates the presence of *L. monocytogenes* in frozen vegetables and the processing environment, representing a potential hazard to public health. Drains and floors are the main points of contamination among the examined processing environment sites. Most of the *L. monocytogenes* isolates belonged to serotype 1/2a-3a and were identified as ST7 and were linked to samples obtained from floors and drains, as well as other types of n-FCS, but also to a positive sample obtained from the final frozen product. This result highlights the role that n-FCS play in the spread of *L. monocytogenes* contamination to the final product, particularly in areas surrounding the freezing tunnel, where most of the contamination was concentrated. Further studies are needed to monitor the transmission pathways of *L. monocytogenes* in frozen vegetable plants and to implement corrective actions to prevent the transfer of this pathogen from the environment to the final products.

## Figures and Tables

**Table 1:** Description of the zones, location, sampling site, number of samples, presence/absence of *L. monocytogenes* and isolate identification.

Zone	Sample Type	No. of Samples	Present/Absent and Number of Positive Samples	Isolate Identification
**FCS**				
	Conveyor-belt before blanching	1	+ (1)	Lm-1
	Blanching (surface of blanching tank)	3	-	
	Cooling bath (water)	1	-	
	A shovel used in the cooling bath	1	+ (1)	Lm-5
	Cooling shower (surface of the sprinkles and conveyor belt)	4	-	
	Conveyor belt for manual inspection	2	-	
	Slicer	6	-	
	Conveyor-belt from the slicer to the freezing tunnel	2	-	
	Freezing tunnel (surface)	5	+ (2)	Lm-6/Lm-8
	A shovel used in the freezing tunnel	1	-	
	Conveyor-belt after the freezing tunnel	2	-	
	Packaging (surface of the packaging machine)	4	-	
	**SubTotal**	**32**	**4**	
**n-FCS**				
	Conveyor-belt before blanching	1	-	
	Blanching	1	-	
	Cooling bath	2	-	
	Cooling shower	4	-	
	Floor and drains around the cooling shower	8	-	
	Floors and drains around the conveyor belt for manual inspection	7	+ (3)	Lm-12/Lm-15/Lm-16
	Slicer	2	-	
	The floor around the conveyor belt from the slicer to the freezing tunnel	2	+ (2)	Lm-18/Lm-19
	Surfaces around the freezing tunnel	3	-	
	Freezing tunnel	2	+ (2)	Lm-20/Lm-21
	Floor and drains around the freezing tunnel	5	+ (2)	Lm-23/Lm-24
	**SubTotal**	**37**	**9**	
**Product**				
	Raw material	3		
	Unpacked final product	3	+ (1)	Lm-26
	Packed final product	3		
	**SubTotal**	**9**	**1**	
	**Total**	**78**	**14**	

**Table 2:** Presence and prevalence of *L. monocytogenes* in the frozen vegetable processing plant.

	*Listeria monocytogenes*			
Sample Type	Positive after Enrichment	Positive by Genedisc	Positive after OCLA/MALDI-TOF	Prevalence * (%)
**FCS**	11/32	9/11	4/9	12.5% (4/32)
**non-FCS**	17/37	15/17	9/15	24.3% (9/37)
**Product**	6/9	3/6	1/3	11.1% (1/9)
**TOTAL**	34/78	27/34	14/27	17.9% (14/78)

* Prevalence of confirmed positive among the tested samples in each sample type.

**Table 3:** Characterisation of *L. monocytogenes* isolates at different zones of the frozen vegetables plant.

Serogroup (Serotype)	Isolate Identification	Sequence Type (MLST)	Clonal Complex (MLST)	Complex Type (cgMLST)
IIa (1/2a and 3a)	Lm-1	8	CC8	-
IIa (1/2a and 3a)	Lm-5	7	CC7	-
IIa (1/2a and 3a)	Lm-15	7	CC7	-
IIa (1/2a and 3a)	Lm-16	7	CC7	-
IIa (1/2a and 3a)	Lm-20	7	CC7	-
IIa (1/2a and 3a)	Lm-21	7	CC7	-
IIa (1/2a and 3a)	Lm-23	7	CC7	
IIa (1/2a and 3a)	Lm-24	7	CC7	
IIa (1/2a and 3a)	Lm-26	7	CC7	
IIb (1/2b, 3b, and 7)	Lm-6	5	CC5	7746
IIb (1/2b, 3b, and 7)	Lm-8	5	CC5	7746
IIb (1/2b, 3b, and 7)	Lm-18	87	CC87	6480
IIb (1/2b, 3b, and 7)	Lm-19	87	CC87	6480
IIb (1/2b, 3b, and 7)	Lm-12	Unknown	-	3714

**Table 4:** Allelic distance detected among each pair of strains of the fourteen *L. monocytogenes* isolates.

	Lm-12	Lm-6	Lm-8	Lm-5	Lm-15	Lm-16	Lm-19	Lm-21	Lm-23	Lm-24	Lm-26	Lm-1	Lm-18	Lm-20
**Lm-12**	0	1065	1077	1047	1082	1627	1616	1618	1595	1627	1619	1614	1604	1596
**Lm-6**	1065	0	1103	1074	3	1587	1580	1581	1560	1589	1583	1580	1566	1560
**Lm-8**	1077	1103	0	1	1120	1622	1612	1617	1591	1623	1616	1612	1599	1596
**Lm-5**	1047	1074	1	0	1095	1578	1566	1572	1550	1576	1574	1570	1557	1550
**Lm-15**	1082	3	1120	1095	0	1606	1596	1600	1575	1608	1600	1598	1581	1576
**Lm-16**	1627	1587	1622	1578	1606	0	1240	1235	1223	1247	1236	1235	1229	1225
**Lm-19**	1616	1580	1612	1566	1596	1240	0	0	1	2	2	1	4	1
**Lm-21**	1618	1581	1617	1572	1600	1235	0	0	1	2	2	1	4	1
**Lm-23**	1595	1560	1591	1550	1575	1223	1	1	0	4	3	2	6	2
**Lm-24**	1627	1589	1623	1576	1608	1247	2	2	4	0	4	3	6	3
**Lm-26**	1619	1583	1616	1574	1600	1236	2	2	3	4	0	3	6	3
**Lm-1**	1614	1580	1612	1570	1598	1235	1	1	2	3	3	0	5	2
**Lm-18**	1604	1566	1599	1557	1581	1229	4	4	6	6	6	5	0	4
**Lm-20**	1596	1560	1596	1550	1576	1225	1	1	2	3	3	2	4	0

## Data Availability

The data presented in this study are available on request from the corresponding author.
